# The Systemic Assessment Clinic, a Novel Method for Assessing Patients in General Adult Psychiatry: Presentation and Preliminary Service Evaluation

**DOI:** 10.1007/s10597-020-00694-5

**Published:** 2020-08-01

**Authors:** Maria Grazia Turri, Stephen Merson, Sue McNab, Ruth E. Cooper

**Affiliations:** 1 Oxford Health NHS Foundation Trust, Warneford Hospital, Oxford, OX3 7JX UK; 2 Centre for Psychiatry, Wolfson Institute for Preventative Medicine, Queen Mary University of London, Charterhouse Square, London, EC1M 6BQ UK; 3 East London NHS Foundation Trust, Newham Centre for Mental Health, London, E13 8SP UK; 4 Newham Centre for Mental Health, Unit for Social and Community Psychiatry, Queen Mary University of London, London, E13 8SP UK

**Keywords:** Psychiatric assessment, Systemic family therapy, Systemic assessment, Service evaluation

## Abstract

The traditional model of psychiatric assessment and diagnosis can be criticised as reductive. We developed an innovative model for psychiatric assessment of adult patients referred to our adult mental health team, the Systemic Assessment Clinic, incorporating the principles and techniques of systemic family therapy and dialogical practice into standard psychiatric assessment. We conducted a service evaluation, comparing prospective use of mental health services for patients assessed either in the Systemic Assessment Clinic or in standard assessment. Patients assessed in the Systemic Assessment Clinic had more favourable outcomes than those in standard assessment: they were significantly less likely to need multiple follow-up treatment appointments with a psychiatrist and to be re-referred to mental health services once discharged, indicating reduced healthcare costs. Satisfaction rates for participants attending the systemic assessment clinic were high. Our service evaluation gives preliminary evidence that the Systemic Assessment Clinic could be a potential new model for psychiatric assessment; further evaluation is warranted in a randomised controlled trial.

## Introduction

The majority of adult patients referred to mental health services receive an initial assessment by one or two clinicians working as part of a multidisciplinary team. The assessment is the first encounter between mental health clinicians and patients, and it is used to determine the diagnosis, the care needs, and the treatment options for each patient. It is also of paramount importance for establishing a good relationship with patients so that they engage with the clinical team and cooperate with the care pathway which supports their treatment and recovery. The assessment usually follows a standard psychiatric interview. Questions are centred on current presentation and symptomatology and are mainly intended to elicit a DSM or ICD diagnosis. The standard interview comprises some items related to the patient’s personal and family history, but these generally elicit relatively ‘closed’ responses, asking questions such as ‘was your childhood happy’, and ‘what sort of family were you raised in’ (Semple and Smyth 2009). Family history is often limited to the presence or absence of psychiatric conditions in relatives (Cooper and Oates 2009). Typically, patients attend their assessment alone, while family members may be interviewed separately, to collect collateral information, aimed at expanding on or clarifying some points discussed during the patient’s assessment.

This traditional model of psychiatric assessment and diagnosis has been criticised in that it can reduce complex human experiences to categorical ‘illnesses’, implying a biological cause with psychiatric medication the first-line treatment (Allsopp et al. 2019). In line with this, the validity of the diagnostic system has been questioned by neuroscience research, which has highlighted its shortcomings in predicting treatment response and identifying correlates of pathophysiological dysfunction (Goldberg 2015; Insel et al. 2010; Thibaut 2018). There have been calls for psychiatric assessments to be changed and instead of focusing on symptoms, a narrative of a person’s life should be created taking into account the difficulties they have faced and their response to these difficulties, with symptoms viewed as an understandable response to often adverse environments (Johnstone and Boyle 2018).

Our (MGT and SM) experience within a systemic family clinic alerted us to the potentiality of addressing these concerns by incorporating dialogical practice and the systemic family therapy approach into the psychiatric assessment. Systemic family therapy is a branch of psychotherapy which includes the active participation of families and carers in discussions and decisions about the person they care for and about. Dialogical practice emphasises the clinicians’ responsiveness to others’ contributions and decentres their role as experts, in order to foster “a common ground for mutual understanding and growth” (Mikes-Liu 2015, p. 214). In involving the family and/or significant others and focusing on the context in which a person's mental health difficulties have emerged, a shared understanding of the difficulties a patient may have experienced is created and potential solutions are derived from considering the whole ‘system’ rather than being solely focussed on the individual patient. There is considerable evidence that systemic family therapy is effective for a wide range of mental health difficulties (Carr 2009, 2014). In the United Kingdom, guidelines by the National Institute for Health and Care Excellence (NICE) recommend the use of family therapy for a range of adult mental health disorders (NICE 2016) and the Department of Health (DoH) has issued policies to promote working in partnership with families such as the establishment of carers’ assessments (DoH 1999), the Think Family strategy (Cabinet Office 2008), and the Triangle of Care (Worthington and Rooney 2010). The latest strategy document for mental health from the DoH (DoH 2011) recognises that families and carers too often report being ignored by health care professionals, despite having detailed knowledge and insight, and being best placed to advise and contribute to the recovery of the relative or friend they are caring for.

Dialogical practice has come to the fore with the implementation of Open Dialogue, a family and social network approach to mental healthcare successfully practiced in Finland and currently being trialled in various other countries globally including the USA and UK (Razzaque and Stockmann 2016). Open Dialogue involves an integrated treatment system that requires a reconfiguration of the whole mental health service delivery and the training of all staff in the specifics of the approach (Olson et al. 2014). At the time of our project, Open Dialogue had not yet been contemplated as an alternative to standard treatment in the UK and to this day, it is only being implemented in a selected number of mental health services that are partaking in a national research trial. We certainly did not have the resources or the knowledge to implement Open Dialogue. However, dialogical practice was an established component of the practice of the systemic family clinic where we trained.

Systemic family therapy is a discipline which has historically been very dynamic in expanding its theoretical ground and adapting its practices to the pressures and constraints of specific health settings (Flaskas 2010; Hartman and De Courcey 2015). Within acute and secondary mental health services, such as those where this research project was carried out, systemic clinics are not uncommonly staffed by practitioners who, having an interest in family therapy but no formal qualification, work under supervision of consultant family therapists (Hartman and De Courcey 2015). Such adapted practice provides for the training of non-family therapists in system and family based approaches, while allowing for the running of additional clinics by a specialist service which is often restricted to a small number of highly qualified family therapists. The family therapy service where we practiced had incorporated dialogical practice within their systemic therapy approach (Wilson 2015).

In the context of a pilot service redesign, we created a new model for psychiatric assessment on the basis of the ‘practice-based knowledge’ that we developed while being part of the team running the systemic family therapy clinic (Mitchell 2011). We called our innovative approach to psychiatric assessment, the Systemic Assessment Clinic (SAC). Following the model of systemic family therapy, the SAC emphasised the importance of family and other social relationships for understanding the causes, context, and solutions to mental health difficulties (Gorell-Barnes 2004). By foregrounding dialogical practice, we focused our interview style on turning problems (or what in psychiatric parlance may be called the ‘presenting complaint’) into questions. These were used to instigate a dialogue involving the patient and the members of the family/social network whom the patient brought with them. Ultimately, the aim of the assessment was to engage all participants, including ourselves as professionals, in achieving a shared understanding of the problems to be assessed, and in generating shared answers and solutions (Garavan 2013). Not only does the SAC offer an assessment which is much less pre-determined by professional expectations and much more attuned to the needs and perspectives of the patient and their family, but it gives rise to a mutual responsibility to implement the agreed solutions. This leads to the effective mobilization of the patient’s own resources and those of their family and social network (Garavan 2013).

Here we describe the SAC as an innovative model of psychiatric assessment and we present the results from a service evaluation of the SAC compared to standard psychiatric assessment.

## Methods

### Setting

The study was conducted in a UK general adult mental health secondary care service which assesses and treats patients who are referred from primary care physicians. The service provides both outpatient (community) and inpatient (hospital) care. The SAC was accessed by both inpatient and outpatient service users. We focussed the service evaluation on patients who had had no previous contact with psychiatric services. New inpatients had usually had previous contact with psychiatric services either via the outpatient team or through a different route of access, such as a Mental Health Act assessment. This meant that the scope of the service evaluation was restricted to the SAC outpatient service.

### The Systemic Assessment Clinic (SAC)

The SAC is an innovative model for assessing psychiatric patients referred to mental health services as an alternative to standard assessment. Between 2010 and 2014 a SAC was run by a consultant psychiatrist (SM) who co-conducted assessments with a family therapist (SMcN) or with a psychiatrist and registrar in psychotherapy (MGT) on alternate weeks. MGT is not a qualified family therapist but she had considerable experience in family therapy, having been part of a systemic family therapy team for 4 years during her psychiatric training. SMcN, the only qualified family therapist working in the SAC, supervised the clinic weekly for the first year, and subsequently co-ran the clinic on alternate weeks as well as providing ad hoc supervision for families seen by MGT and SM alone.

The SAC was offered to psychiatric patients at various stages of their clinical care: some were new inpatients, some were outpatients who were already under the care of the mental health team and needed a review of their care needs, some were re-referred by their primary care physician (General Practitioner (GP)) after previous episodes, while a minority were patients who were newly referred and had no previous history of contact with psychiatric services. This latter subgroup was the sample for our service evaluation.

The assessment framework consisted of an initial meeting and at least one follow-up session, although in some cases more than one follow-up sessions were offered. The overall number of assessment sessions offered therefore ranged from 2 to 4. Each initial assessment session lasted 1.5 h and was based on the framework of a systemic family therapy assessment to which the patients were invited to bring members of their family and any significant others. The first follow-up session was typically one-hour long and was scheduled within 4 weeks of the initial appointment. Before the first assessment visit, the consultant made contact with the referred patient to explain about the clinic and invite them to bring along members of their family, close friends or carers. The assessment concluded with a letter to the primary care physician (always copied to the patient) summarising the content of the conversations and outlining the agreed care plan. Offer of further treatment and referrals to other services were carried out as per routine care.

In the SAC, we applied a systemic and dialogical practice approach to psychiatric assessment, which implied two broad changes to the standard assessment model. First of all, patients were invited to bring family members or any other person who is significant to them to the assessment meetings and the assessment took place with all those present. Secondly, the assessors adopted a dialogical interviewing style. Compared to standard assessment, this was characterised by the use of open questions to elicit the problem (presenting complaint) from the narrative of the patients’ and wider networks′ experience, such as:

- What do you think the problem is?
- What has made a positive or negative impact on the problem?
- What would be a good outcome of today’s meeting?
- How would you know that the problem had got better?

These questions were asked to all those present in the assessment, and not solely focused on the patient. Themes that emerged from the open questions, such as family disagreements about certain behaviours, or confusion about a diagnosis guided the development of the conversation within the assessment. The assessing clinicians strived to give equal attention to each participant’s narrative and their style of facilitation was characterised by noting points of concordance and discordance and occasionally by offering alternative narratives to be confronted and discussed on the par to those of the patient and family members.

Because the dialogue was constructed around the themes that were brought forward by the patient and significant others, every interview was unique. Some patients and families, for instance, emphasised current psycho-social stressors, such as moving to university or loss of a job, others were more interested in discussing symptoms and choices of medication, while others referred to difficult family dynamics (see ‘personal experience’ for an account of one patient). Questions necessary to cover obligatory elements of the standard assessment, such as risk-assessment and determining ICD-10 diagnosis, were interweaved within the open dialogue that was established during the assessment, or if this was not possible they were asked at the end of the assessment. The aim of each session was to give everyone an opportunity to contribute their views, in order to reach an agreed understanding of the situation and a shared plan of action (Garavan 2013).

### Standard Assessments

With regards to standard assessments, the consultant’s (SM) team’s common practice during the time of the study was as follows: standard assessments typically lasted one hour and were carried out jointly by two professionals, of whom at least one was an experienced clinician such as a psychiatrist, a community psychiatric nurse or a social worker. Patients were invited to the assessment through a letter, which did not mention the possibility of bringing other people to the assessment. If patients arrived at the assessment accompanied, they were asked whether they wanted their family members or friends to join, but the general expectation was that at least a part of the assessment interview would be conducted with the patient alone. The assessment generally followed a standard outline and the main areas explored were: symptoms, past psychiatric history, response to previous treatments, and family history of mental health conditions (Cooper and Oates 2009).

### Ethics

As this was a service evaluation ethical approval was not required.

### Participants

For the scope of this service evaluation we selected only those patients who presented to psychiatric services for the first time; these constituted a minority of the total number of patients seen in the SAC and they all presented as outpatients. The age of participants was within the remit of the mental health team, i.e. adults of working age between 18 and 65 years old.

Because the SAC had a minimal capacity of one assessment per week, only a proportion of patients newly referred to the team were seen in the SAC while the others were seen in standard assessment. There were no criteria for choosing the patients who would be assigned to the SAC: this was done pragmatically according to whether there was an available SAC slot when the referral was received. We adopted a non-selection strategy because we realized that it would have been impossible to pre-determine which patients would benefit more from the SAC approach, as there is no evidence-base to establish this. Moreover, referral letters from primary care physicians do not usually contain sufficient information to suggest, for instance, if family dynamics are relevant to the current presentation or if family or friends are involved with the patient’s care.

### Data Gathering

22 consecutive outpatients who were newly referred for a psychiatric assessment and were seen in the SAC from late 2010 to 2013 were included in this service evaluation. For the scope of comparison with treatment as usual, 21 outpatients who were newly referred to the same mental health team during the same time period, and seen in standard assessment, were included in the study. Patients to be included in the comparison group were picked from the team’s assessment diary according to the following procedure: of the list of patients newly-referred to the team and with no previous contact with mental health services, 22 were selected for whom the date of assessment matched more closely that of a patient assessed in the SAC. There was no knowledge of the patient’s diagnosis or clinical history when the selection was made. Of the 22 selected, one had to be excluded because it was later discovered that they had previous contact with psychiatric services.

For all study subjects, we surveyed the mental health records from the time of assessment to the end of 2014. All episodes of use of mental health services were recorded. We call ‘follow-up period’ the length of time for which the survey of mental health records could be carried out. As patients included in the study were assessed at different times between late 2010 and 2013, the ‘follow-up’ period varies from 1 to 4 years. For instance, for a patient assessed in July 2011, the follow-up period is 3.5 years. Our follow-up data are as follows: for 7 subjects in each group, we have follow-up data for three-to-four years; for 6 subjects in the SAC and 5 subjects in the standard assessment group we have follow-up data for two-to-three years; and for 9 subjects in each group, we have follow-up data for one-to-two years. Long follow-up periods are important to the study, as the outcome measures of the service evaluation were the extent of use of mental health services after assessment. The follow-up care that was received by patients encompassed: follow-up care with the mental health team (appointments with community psychiatric nurses, social workers, occupational therapists, or support workers, follow-up with tertiary services), treatment from a psychiatrist, referral to psychology or psychotherapy, or a combination of these. We also recorded time to discharge and rates of re-referral after discharge.

Subjects (patients and accompanying significant others) who attended the SAC were asked to rate their satisfaction with the assessment by completing a short questionnaire. This questionnaire was adapted from the Session Rating Scale (Duncan et al. 2003) and measured the degree of satisfaction with the overall assessment and with the experience related to the assessment, in particular ‘feeling that one had been heard, respected and understood’, and ‘feeling that the session had focused on topics that were important to the individual’. Items were scored using a Likert scale. We gave the questionnaires out at the end of the first assessment session and we asked participants to return them to the clinic reception so that respondents would remain anonymous. It was not possible to collect satisfaction data for patients participating in standard assessments.

### Data Analysis

Data collected for all participants were pooled within the two groups and compared. Differences between the groups were calculated using t-tests (for continuous data), Chi square or Fisher’s Exact (if frequencies were ≤ 5) tests (for categorical data) using STATA (version 14.2). Ratings of satisfaction were pooled across the whole group of participants in the SAC and also pooled according to two subgroups: those obtained from referred patients and those obtained from non-patients (family members, friends or carers) participating in the assessment session.

## Results

### Sample Characteristics

There were no differences in gender and age between the two groups. The distribution of diagnoses differed slightly between the groups with patients seen in the SAC tending to have more psychotic disorders and severe depression, while there were more patients with moderate depression or anxiety disorder in the standard assessment group, however these differences were not statistically significant (Table 1).

**Table 1:** Demographic and mental health service use data for patients participating in the Systemic Assessment Clinic (SAC) and for those in standard assessment

		SAC (total n = 22) (%)	Standard assessment (total n = 21) (%)	*p* value
Gender	Female	14 (64)	14 (67)	0.84
	Male	8 (36)	7 (33)	–
Age	Mean (SD)	39 (14.0)	41 (11.4)	0.5
	Range	20–66	22–59	–
Diagnosis	Psychosis/ schizophrenia	6 (27)	3 (14)	–
	Bipolar illness without psychosis	1 (5)	2 (10)	–
	Non psychotic disorder of overvalued ideas (e.g. OCD, eating disorder)	4 (18)	3 (14)	–
	Chronic and severe personality disorder	2 (9)	3 (14)	–
	Severe depression	7 (32)	4 (19)	–
	Depression or anxiety moderate	2 (9)	6 (29)	0.5
Follow-up care				
Follow-up care with the mental health team	None	15 (68)	10 (48)	0.27
	< 6 months	3 (14)	2 (9)	–
	1 + years	2 (9)	7 (33)	–
	Long-term follow-up with tertiary mental health service, e.g. early intervention for psychosis service; eating disorder service	2 (9)	2 (9)	–
Meetings with psychiatrist for treatment	None	13 (59)	6 (29)	.003**
	1–2	7 (32)	3 (14)	–
	≥ 3	2 (9)	12 (57)	–
Referral to psychology or psychotherapy	No	15 (68)	11 (52)	0.29
	Yes	7 (31)	10 (48)	–
Discharge	Immediately after assessment	4 (18)	3 (14)	0.29
	Within 6 months of assessment	7 (32)	3 (14)	–
	Between 1 to 2 years from assessment	3 (14)	8 (38)	–
	After 2 or more years from assessment	8 (36)	7 (33)	–
Re-referrals to mental health services after discharge	No	21 (95)	12 (57)	.004**
	Yes	1 (5)	9 (43)	–

Data are given as absolute numbers and as percentages

**p < .01, n = number of participants

### Assessment Composition

Table 2 shows whether patients attended the assessment alone or with family or significant others. As expected, in the majority of cases patients seen in the SAC were seen with either family or friends, while patients seen in standard assessment were typically seen alone. There were however a few exceptions in both cases. When patients attended alone in the SAC, the systemic and dialogical style of interviewing was maintained, and it included asking open questions about the hypothetical opinions of family members or friends who were not in the room. It should also be noted that in two cases of the SAC, the referred patient did not attend, but he/she gave permission for his/her parents to be interviewed in their absence. In the SAC a consultant psychiatrist (SM) was always present. Due to limitations in the medical records it was not possible to extract the exact number as to the presence or absence of a psychiatrist in the standard assessment. Specifically, if the assessment letter had been written by a psychiatrist, it was certain that a psychiatrist had been part of the assessment team. However, when the assessment letter was written by another member of the team, it was not always possible to ascertain whether a psychiatrist had been part of the assessing team. It is reasonable to suppose that in standard assessment a psychiatrist was not invariably present as other members of the multidisciplinary team can at times assess a patient without direct input from a psychiatrist.

**Table 2:** Composition of the assessment meeting for the Systemic Assessment Clinic (SAC) and for standard assessment

	SAC (total n = 22) (%)	Standard assessment (total n = 21) (%)
Patient seen with family and/ or significant others	18 (82)	3 (14)
Patient seen alone	4 (18)	18 (86)

Data are given as absolute numbers and as percentages

n = number of participants

### Outcomes

The length of treatment within mental health services for both groups was comparable. A minority of patients were discharged back to the GP immediately after assessment (18% in the SAC and 14% in standard assessment) (Table 1). The remaining patients were referred for a range of treatment options, including follow-up treatment with a psychiatrist, follow-up treatment with the mental health team (usually delivered by a psychiatric nurse, social worker, occupational therapist or support worker), referral to psychology or psychotherapy, or referral to a tertiary service within mental health, such as the eating disorder service or the early intervention service for psychosis.

The number of patients requiring follow-up with a psychiatrist for treatment (as opposed to assessment) was higher in the standard assessment group (Fisher’s exact p = 0.003). A higher proportion of patients seen in the SAC did not require treatment with a psychiatrist (59% in the SAC vs 29% in standard assessment) while a higher proportion of patients seen in standard assessment required 3 or more treatment follow-ups with a psychiatrist (57% in standard assessment vs. 9% in the SAC). Interestingly, the 6 subjects in the standard group who did not require any follow-up treatment with a psychiatrist, had seen a psychiatrist at assessment (Table 1). This indicates that patients in standard assessment who may not have been seen by a psychiatrist as part of the assessment, were subsequently referred to a psychiatrist for treatment.

Two patients in both groups were referred to a tertiary mental health team for treatment (e.g. an eating disorder service or an early intervention for psychosis service). Of the remaining subjects, the number of patients requiring follow-up care with the mental health team (excluding psychiatry) was again higher in the standard assessment group: 9 (42.5%) subjects versus 5 (23%) for the SAC group, but this difference was not statistically significant (Table 1).

Referrals for psychotherapy or psychological treatment, including family therapy, occurred for 10 (48%) patients in the standard group versus 7 (31%) in the SAC group, a difference which is not statistically significant, additionally showing that patients seen in the SAC were not more likely to be referred for a psychological intervention (Table 1).

Rates of discharge between both groups were relatively comparable. However re-referrals following discharge differed significantly between the two groups. In the SAC group, only 1 (5%) subject was re-referred for a second time to mental health services after discharge, compared to 9 (43%) subjects in the standard group (Fisher’s exact p = 0.003). For these subjects, 4 (19%) were re-referred once and 5 (24%) were re-referred twice during the follow-up period (Table 1).

Table 3 shows the rates of satisfaction with the SAC in 48 participants, of which 19 were identified patients and 29 non-patients. Overall satisfaction was high for most participants, with only 2 (4%) participants (both patients) indicating low rates of satisfaction. 83% of participants were highly satisfied with the style of assessment and the remaining were at least moderately satisfied.

**Table 3:** Rates of satisfaction with the SAC as reported by patients and non-patients

	Overall sample (total n = 48) (%)	Patients (total n = 19) (%)	Non-patients (total n = 29) (%)
Overall satisfaction with assessment			
High (6–7)	41 (85)	15 (79)	26 (90)
Moderate (4–5)	5 (11)	2 (10)	3 (10)
Low (1–3)	2 (4)	2 (10)	0 (0)
Satisfaction with the style of assessment			
High (6–7)	40 (83)	15 (79)	25 (86)
Moderate (4–5)	8 (17)	4 (21)	4 (14)
Low (1–3)	0 (0)	0 (0)	0 (0)
‘I have felt heard, understood and respected’			
High (6–7)	40 (83)	12 (63)	28 (97)
Moderate (4–5)	7 (15)	6 (32)	1 (3)
Low (1–3)	1 (2)	1 (5)	0 (0)
‘We talked about what I wanted to talk about’			
High (6–7)	38 (79)	13 (69)	25 (86)
Moderate (4–5)	9 (19)	5 (26)	4 (14)
Low (1–3)	1 (2)	1 (5)	0 (0)

Data are given as absolute numbers and as percentages

n = number of participants

## Discussion

### Principal Findings

This service evaluation is one of the first to investigate outcomes for a form of psychiatric assessment that is different from the ‘standard’ assessment, and to provide comparative data. We found that patients newly referred to mental health services and assessed in the Systemic Assessment Clinic (SAC), a novel assessment method which incorporates principles and techniques of systemic family therapy and dialogical practice, had more favourable outcomes in terms of their use of mental health services and the length of care needed from these services, compared to patients assessed in standard psychiatric assessment. This provides preliminary data that the SAC could be a promising alternative to the standard psychiatric assessment which warrants further investigation in a randomised controlled trial.

Our most striking result was that nearly all patients assessed in the SAC were *not* re-referred back once discharged, with one single exception. The situation was very different in the standard group, where nearly half the patients were re-referred back after discharge and of these, half were re-referred back twice during the follow-up period. Despite our approach being limited to the assessment, with standard care options the same for both groups, we hypothesise that the dialogical approach at assessment and the involvement of the patient’s system were instrumental in formulating the presenting problem and identifying the best solutions (Garavan 2013). For example, a patient seen in the SAC was affected by severe depression. He attended with his wife and reported an extensive history of childhood abuse, which they considered partly responsible for his current symptoms. At face value, we might have judged it appropriate to refer him for psychotherapy. However, our dialogical approach led us to discover that he felt supported by his wife and preferred to talk through his experiences and flashbacks with her, to which she agreed. Instead, they welcomed the idea of medications to help with his sleep and reduce his anxiety. Another example of how the SAC approach led to unexpected outcomes and resolution is given in the ‘personal experience’ section.

While a minority of patients in both groups needed referral to tertiary mental health services for long term care, for the majority of patients who remained within secondary mental health care, those assessed in the SAC were significantly less likely to need multiple follow-up treatment sessions with a psychiatrist. The SAC was staffed with highly qualified clinicians: a consultant psychiatrist with many years of experience (SM), a psychiatrist and specialty registrar in psychotherapy who qualified as consultant in 2011 (MGT) and a consultant family therapist with many years of experience (SMcN). While in the context of the multidisciplinary team, it may seem counterintuitive to have highly skilled clinicians assess all patients, our results suggest that the SAC may reduce the need for follow ups with a psychiatrist at the treatment stage and reduce rates of re-referral and therefore overall costs of psychiatric care for a substantial proportion of cases.

The overall satisfaction with the assessment process and with the style of assessment was generally very high among both patients and non-patients attending the SAC. The majority of participants agreed that they felt understood and that they could talk about what was important to them. Although a minority of patients (4% in our sample) were not satisfied with the assessment, our data show that the SAC is generally well-liked by both patients and non-patients attending. This result ties in with our personal experience of running the clinic, with participants often expressing a sense of relief and gratification at the end of sessions. However it is important to note that satisfaction data were not collected for standard assessments, so it was not possible to compare satisfaction levels of the SAC to standard assessment.

## Limitations

The results of this study are based on a service evaluation which is subject to a number of limitations. The evaluation was conducted in one location with a small sample size. We did not have access to purposely measured outcome data as the service evaluation was conducted post-hoc, with data primarily collected from medical records. The data collection was unblinded and data were collected by one of the clinicians who ran the SAC (MGT). The potential for bias can therefore not be ruled out. Due to heterogeneity in the medical records it was difficult to extract some of the required data (such as the presence or absence of a psychiatrist in the standard assessments). Moreover, our preliminary results cannot establish what elements of the SAC may determine its effectiveness; further studies are needed to investigate whether it is the dialogical and systemic framework which underpins its outcomes, or rather the longer duration of the assessment, the fact that it is conducted by highly qualified professionals, the presence of family members or friends, or a combination of all these elements. Given these limitations these results should be viewed as preliminary, warranting further investigation within a randomised controlled trial with blinded data collection. The trial should investigate the suggested positive outcomes of the SAC, testing its effectiveness and cost-effectiveness across various settings and with a larger sample.

## Conclusions

In recent years there has been increasing recognition that working with families is central to the treatment of mental health conditions. There is considerable evidence that family interventions are effective for a wide range of mental health difficulties (Carr 2014; Razzaque and Stockmann 2016; Stratton 2010) and are cost effective in comparison to alternative treatments (Crane 2008). Family intervention has been reported to have particularly positive outcomes in psychosis (Seikkula et al. 2011). Alongside this and since we conducted this study, an alternative to traditional psychiatric diagnosis, ‘The Power, Threat, Meaning Framework’ has been established (Johnstone and Boyle 2018). In line with the approach used in the SAC, this framework suggests that through asking open questions such as ‘What has happened to you? How did it affect you? ‘What did you have to do to survive?’, a narrative should be created about a person’s life, the difficulties they have faced, and their response to these difficulties. Psychiatric symptoms can then be viewed as an understandable response to often very adverse environments. However, despite the evidence base and the exhortation of national guidelines and local policies toward greater involvement for families and carers of patients with mental health conditions and guidelines for alternatives to traditional psychiatric diagnosis, these aspirations have not readily transferred into routine clinical practice, at least in the United Kingdom.

Feedback from families shows that family members and carers typically feel excluded by mental health services (Stanbridge and Burbach 2014). In a study investigating patients’ experiences of psychiatric assessment, many participants believed that family and friends could facilitate communication with clinicians at the time of assessment (Bilderbeck et al. 2014). The same study also showed that patients ‘were dismayed by perceived inflexibility in the treatment options that their clinicians offered them or described feeling ‘fobbed off’ by medication, and thereby denied a more thoughtful appraisal of their problem’. Although the study was limited to patients suffering with mood instability, our experience as clinicians indicates that similar attitudes may be common to other psychiatric patients experiencing standard assessment.

The literature shows that early family involvement in mental health treatment has a number of advantages such as clarifying the problem, defining it in the language of the patient and family and mobilising their psychological resources so that they can increase engagement and agency in the process of recovery (Garavan 2013; Hartman and De Courcey 2015; Seikkula and Alakare 2014; Ulland et al. 2014). For instance one study showed that involvement of the family at first contact with psychiatric services significantly reduced rates of unnatural-cause mortality in patients with schizophrenia compared to those with no family involvement (Reininghaus et al. 2015).

The SAC introduces two innovations into standard psychiatric assessment: the involvement of the whole family (or, when this is appropriate, of other persons significant for the patient) on the one hand, and the open style of dialogical practice which does not address a narrow repertoire of signs and symptoms, but increases the focus towards understanding needs, preferences and meaning. We believe that it is the synergic effect of these two factors which underlies the value of the SAC.

This study provides preliminary data which indicates that the SAC could improve patients’ outcomes in terms of service use and re-referral rates and, ultimately, prove a more cost-effective intervention than standard assessment. The most important results of our service evaluation are the much lower rate of re-referrals after discharge and the lower rate of multiple follow-up treatment sessions with a psychiatrist, which in standard practice are often necessary when the problem and its solutions have not been well defined, or when a psychiatrist was not present initially. We think that these positive results are possible because the SAC reaches a more conclusive and authoritative assessment, which signposts patients to the most appropriate service and whose recommendations are followed-through. The active involvement of the patient and their family or friends in the assessment discussion results in patients and families influencing treatment decisions and contributing to a shared ownership of the care-plan, which better aligns with their expectations and needs. Moreover, the assessment discussion is often an opportunity for optimising the use of resources that the families themselves will provide. The positive satisfaction of the patients with the assessment, shows that the SAC model is well-liked by patients and their families and friends, and it is possible that this positive experience may also contribute to further improvement in their engagement with services after assessment. We believe that our study suggests that the value of introducing a systemic and dialogical assessment method for all psychiatric patients needs further investigation, as it seems to improve patients’ outcomes, preventing the revolving-door problems that we see in psychiatry, with patients coming back to services again and again because they have not found a satisfactory resolution to their problem. Further investigation of the SAC in a large randomised controlled trial is warranted.

### Personal Experience: [Maria Grazia Turri]

Mrs B came to see me in my consultant’s outpatient clinic, where I was working as a psychiatrist in training. She had a diagnosis of treatment-resistant depression and I had to switch her current failed antidepressant to a new one, and monitor her progress. She did well for a few weeks, but it did not last long. In passing, I also discovered that her 6 year old daughter was being seen by the children services for enuresis. I think it stuck with me because I felt sorry for them both, but kept doing my job of assessing symptoms and asking the standard questions. I was being a good trainee. A year later I was part of a psychotherapy supervision group where another more senior clinician was seeing Mrs B. Eventually, after gathering additional diagnoses of bipolar II disorder and borderline personality disorder, Mrs B. had been referred for psychotherapy. She disclosed that her husband was forcing her to have sex on a regular basis, in the same bedroom where, she ashamedly admitted, their little daughter (supposedly) slept. While she was accumulating diagnosis after diagnosis, and the child services were wracking their brains to discover the cause of the enuresis, no one (except of course herself, her husband, and the little girl) knew anything about the abuse. It will be said that this is an extreme case. Is it? What about the woman, with bipolar disorder, who comes as a participant in a clinical trial of lithium and reveals for the first time that she was sexually abused by a relative in her adolescence? What about the man, with a diagnosis of schizophrenia, recruited into an antipsychotic trial, who talks ‘matter of fact’ about his parents: his father killed his mother, and burnt the house down, when he was a small child. Perhaps it is the fear of opening Pandora’s box that makes clinicians embrace symptom-based assessment regimes. The fear of the horror behind the patients’ stories justifies the reluctance to engage with the narrative.

And yet, not all stories are as extreme as these ones. George was a young man who had recently been assessed by the psychiatric night team following an incident when he had gone missing from home. His parents had eventually managed to track him down, and they had found him alone in a meadow near their home, allegedly having taken a small overdose of paracetamol. For the past month or so he had been self-harming by superficially cutting his arms and he reported persistent suicidal thoughts and low mood. He was uncooperative with assessment by the night team, and there was some discussion whether he should be assessed under the Mental Health Act. However it was decided, with his parents’ agreement, that he would be ‘kept on watch’ and visited daily by the crisis team, until, a couple of days later, he came for assessment in the Systemic Assessment Clinic (SAC).

George had recently started university in a far away city and that was the time when his low mood had began. What was initially construed as being his difficulty in adapting to his new life, and perhaps the challenges of academic and social demands, became rapidly viewed by his tutors, his parents and himself, more as an illness, possibly depression. He therefore was encouraged to return home before the end of term, but the fact that his condition, if anything, deteriorated further, was seen as confirmation that his problem had not been homesickness, but a genuine mental illness. I leave it to the reader to imagine what may happen next, if the standard assessment framework is followed.

In his first meeting at the SAC, George came with his mother alone. They talked about several things but what stuck out most was the father’s absence and that he had gone to watch rugby in the pub with his friends. As if he did not care about George. We made a suggestion that father may be invited to come to the next meeting, to which all agreed. And he came. In the second session, narratives about leaving the parental home for the first time were confronted. Mother had gone to university and had very much struggled, like George. She had confided in her own mother as a source of support and she described lengthy daily telephone calls that alleviated her anguish. Father had left home to get a job up North, to establish himself in his profession, so that he could secure himself a position that would allow him to marry and start a family. Both had each other when they left home and both were hopeful that at the end of their solitary endeavours, there was a new family home and family life awaiting them.

George instead had broken up with his girlfriend at the time they left high school, where they originally had met, to go to university in cities far apart. Apparently by mutual agreement, they had decided to take some time ‘off’, to give themselves space to form new relationships and to let nature take its course. In the session, it appeared uncertain whether this solution had been truly wished for by George, or at least he was now voicing concern and distress at the thought that he had let go of a cherished relationship. Differently from his parents, for him there was no certain reunion at the end of the solitary journey. Father was the most transformed by this meeting. He had been cold and stern with George, interpreting his current condition as ‘lack of determination’ and even slackness. This had caused tension in the house and arguments between him and George, who, understandably, felt rejected. The family left the session with a promise of mutual support and his father encouraged George to take his time and not feel pressurised to make a decision yet about whether he wanted to return to his studies or not. George was discharged and he did not come back to psychiatric services, at least for the 3 years after assessment.
